# Supplementary material for: Annual Censuses and Citizen Science Data Show Rapid Population Increases and Range Expansion of Invasive Rose-Ringed and Monk Parakeets in Seville, Spain
Source: Animals (Basel). 2022 Mar 8;12(6):677. doi: 10.3390/ani12060677 (PMC8944835; doi:10.3390/ani12060677)
Supplement: Supplementary file 1 [file animals-12-00677-s001.zip › animals-1563608-supplementary.pdf]

Supplementary material

Annual censuses and citizen science data show rapid population increases and range expansion of invasive Rose-ringed and Monk parakeets in Seville, Spain

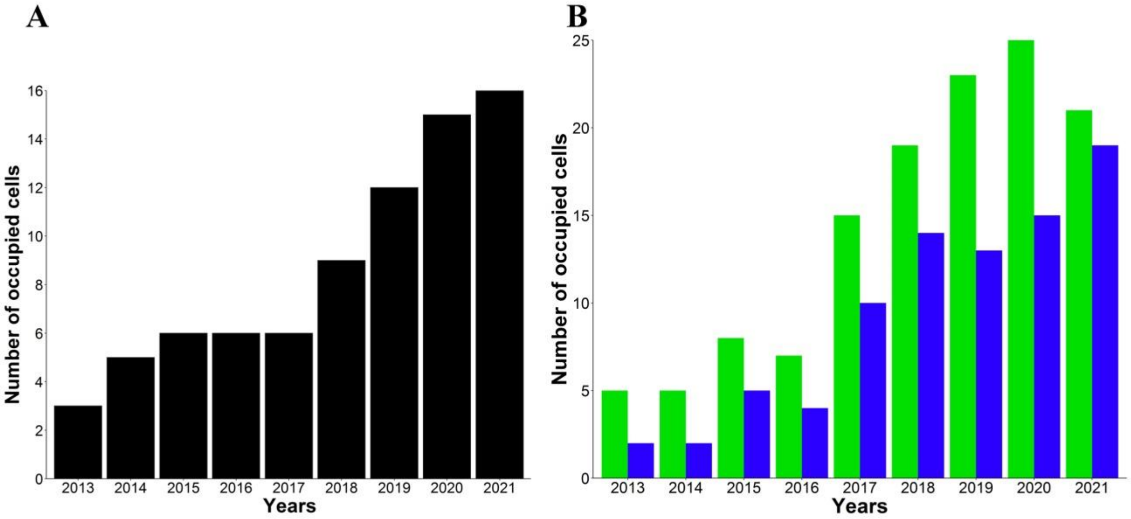

Figure S1. Temporal evolution of the spatial distribution of rose-ringed and monk parakeets in the metropolitan area of Seville (southern Spain). Graphs show the cumulative number of 5x5 km occupied cells over time (2013-2021) regarding A) monk parakeet nests and B) observations of both parakeet species (green bars: rose-ringed parakeets, blue bars: monk parakeets) recorded by citizen science (eBird and Observation).

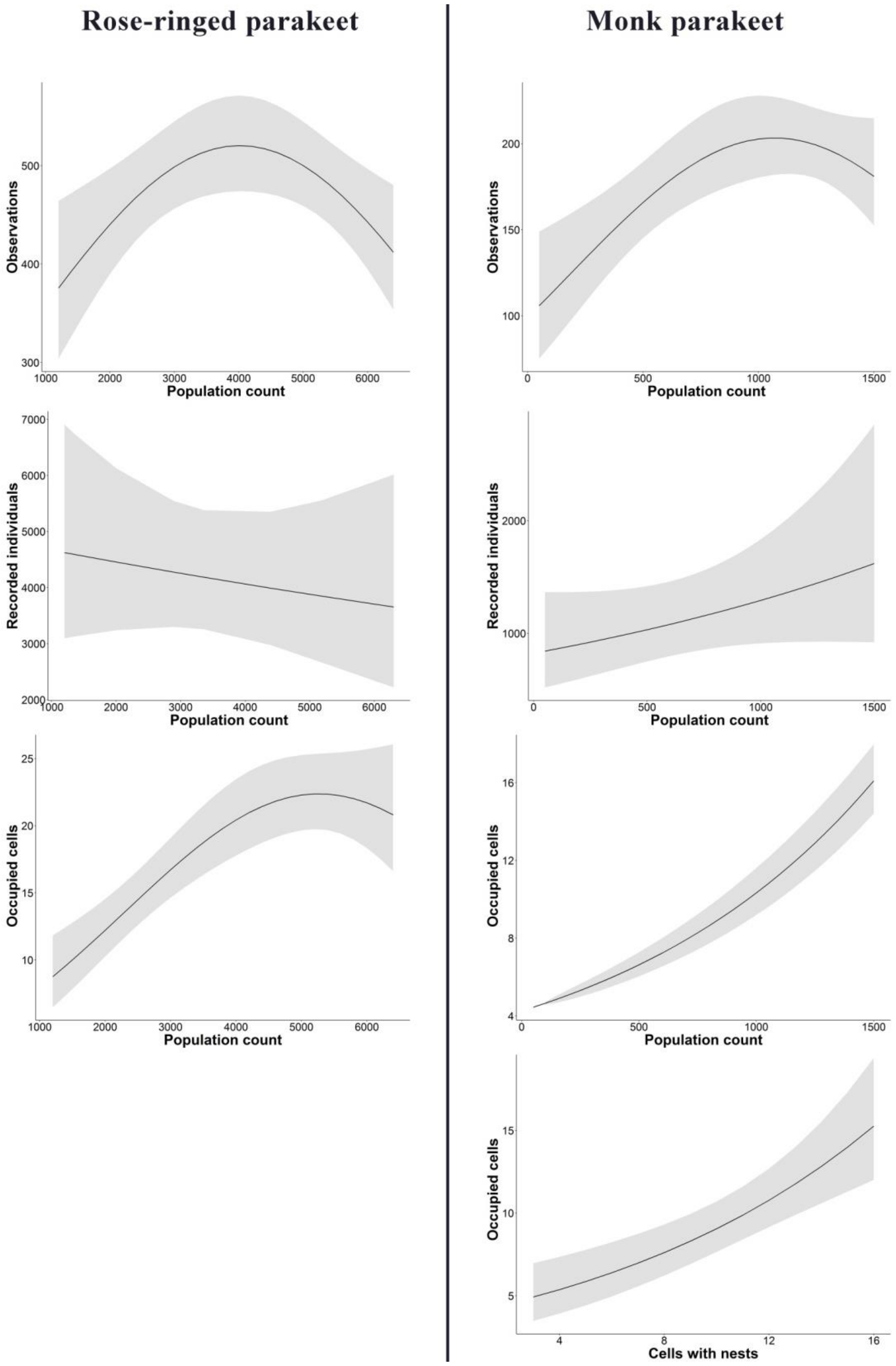

Figure S2. Relationship between the number of observations, the number of recorded individuals, and the number of occupied cells included in the citizen science platforms and our population counts of rose-ringed and monk parakeets and the number of cells with monk parakeet nests. All models included the number of bird observations or the number of cells with bird observations as an offset variable to control for differences in sampling effort over years.

Table S1. Annual population sizes of rose-ringed and monk parakeets established in the metropolitan area of Seville from 2013 to 2021. For the monk parakeet, we estimated the associated 95% CI.

Year	Rose-ringed parakeet	Monk parakeet
2013	1200	70 ± 13
2014		100 ± 19
2015	1367	199 ± 37
2016	2000	372 ± 69
2017	2891	587 ± 108
2018	3360	904 ± 167
2019	4338	1043 ± 192
2020	5185	1307 ± 241
2021	6300	1487 ± 273

Table S2. Generalized linear models (GLM; negative binomial error distribution, log link function) obtained to test the population trend (Survey) of rose-ringed and monk parakeets in the metropolitan area of Seville from 2013 to 2021. Final models were the model with the lowest AIC. dev. expl.: deviance explained (%).

**Rose-ringed parakeet**							
**Model**	**Variable**	**Estimate**	**SE**	***z***	***p***	**AIC**	**dev. expl.**
Survey ~Year	Intercept	-445.22	26.06	-17.08	<0.0001	117.89	97.23%
	Year	0.22	0.01	3.87	<0.0001		
Survey ~Year^2	Intercept	7.96	0.03	250.49	<0.0001	119.73	
	Year	1.59	0.09	17.53	<0.0001		
	Year^2	0.04	0.09	0.4	0.689		
**Monk parakeet**							
**Model**	**Variable**	**Estimate**	**SE**	***z***	***p***	**AIC**	**dev. expl.**
Survey ~ Year^2	Intercept	6.07	0.02	262.44	<0.0001	94.02	99.56%
	Year	3.16	0.08	*39.76*	<0.0001		
	Year^2	-0.77	0.06	-12.11	<0.0001		
Survey ~ Year	Intercept	-800.56	63.9	-12.53	<0.0001	115.6	
	Year	0.4	0.03	12.63	<0.0001		

Table S3. Number of nesting substrates per tree species used by monk parakeets in the metropolitan area of Seville from 2013 to 2021.

Tree species	Family	Total	Percentage (%)
Phoenix dactylifera	Arecaceae	527	73.81
Phoenix canariensis	Arecaceae	106	14.85
Washingtonia robusta	Arecaceae	25	3.50
Washingtonia filifera	Arecaceae	24	3.36
Platanus × acerifolia	Platanaceae	12	1.68
Trachycarpus fortunei	Arecaceae	10	1.40
Yucca gigantea	Asparagaceae	6	0.84
Eucalyptus camaldulensis	Myrtaceae	2	0.28
Ulmus minor	Ulmaceae	2	0.28

Table S4. Linear model (LM), quadratic regression model (QM), and generalized additive model (GAM) obtained to test the temporal trend of the mean annual number of active chambers per nest in a monk parakeet population established in the metropolitan area of Seville from 2013 to 2021. In GAM, the independent variable (Year) is denoted by smooth terms. edf: effective degrees of freedom, Ref.df: reference degrees of freedom used in computing test statistic and the p-values. GAM was significantly better than LM and QM (ANOVA; p <0.013).

**Linear model (LM)**						
**Dependent variable**	**Independent variable**	**Estimate**	**±SE**	***t***	***p***	**R^2**
Number mean of chambers	Intercept	-201.75	52.67	-3.83	0.006	0.64
	Year	0.1	0.02	3.87	0.006	
**Quadratic regression model (QM)**						
**Dependent variable**	**Independent variable**	**Estimate**	**±SE**	***t***	***p***	**R^2**
Number mean of chambers	Intercept	1.95	0.04	45.74	<0.0001	0.85
	Year	0.78	0.13	6.11	0.0009	
	$Year^2$	-0.43	0.13	-3.38	0.015	
**Generalized additive model (GAM)**						
**Dependent variable**	**Independent variable**	**Estimate**	**±SE**	***t***	***p***	**R^2**
Number mean of chambers	Intercept	1.95	0.04	45.93	<0.0001	0.85
		**edf**	**Ref.df**	***F***	***p***	
	Year	1.91	1.99	25.36	0.0014	

Table S5. Models obtained to relate the number of rose-ringed and monk parakeet observations and individuals recorded in the citizen science platforms to our annual population counts (Survey) conducted in the metropolitan area of Seville from 2013 to 2021. The number of bird observations recorded in these platforms was fitted as an offset variable to control for changes in sampling effort over years. We also include models obtained to relate the number of cells with rose-ringed and monk parakeet observations recorded in these platforms to the annual population counts and the number cells with monk parakeet nests (Nests). The number of cells with bird observations recorded in these platforms was fitted as an offset variable to control for changes in sampling effort over years.

**Rose-ringed parakeets**					
**Dependent variable**	**Independent variable**	**Estimate**	**SE**	***z***	***p***
Number of observations	Intercept	-3.46	0.13	-26.49	<0.0001
	Survey	<0.0001	<0.0001	0.52	0.6
Number of observations	Intercept	-3.43	0.04	-82.4	<0.0001
	Survey	0.16	0.12	1.29	0.19
	Survey^2	-0.3	0.11	-2.8	0.005
Number of individuals	Intercept	<-0.0001	<0.0001	-0.62	0.54
	Survey	-1	0.06	-16.97	<0.0001
Number of individuals	Intercept	-1.34	0.13	-10.73	<0.0001
	Survey	-0.24	0.35	-0.67	0.502
	Survey^2	-0.23	0.35	-0.64	0.522
Number of cells	Intercept	-1.45	0.11	-9.41	<0.0001
	Survey	0.0001	0.31	2.92	0.00353
Number of cells	Intercept	-1	0.06	-16.97	<0.0001
	Survey	0.98	0.17	5.28	<0.0001
	Survey^2	-0.41	0.15	-2.69	0.008

**Monk parakeets**					
**Dependent variable**	**Independent variable**	**Estimate**	**SE**	**z value**	**p**
Number of observations	Intercept	-4.35	0.13	-32.45	<0.0001
	Survey	0.0003	0.0001	2.62	0.0237
Number of observations	Intercept	-4.2	0.06	-6.6	<0.0001
	Survey	0.63	0.2	3.2	<0.002

	Survey2	-0.35	0.15	-2.41	0.02
Number of individuals	Intercept	-2.38	0.03	-75.71	<0.0001
	Survey	<0.0001	<0.0001	0.791	0.429
Number of individuals	Intercept	-2.56	0.13	-18.98	<0.0001
	Survey	0.72	0.41	1.77	0.08
	Survey2	-0.62	0.4	-1.53	0.12
Number of cells	Intercept	-2.12	0.14	-14.54	<0.0001
	Survey	0.0009	0.0001	6.63	<0.0001
Number of cells	Intercept	-1.56	0.07	-22.16	<0.0001
	Survey	1.51	0.21	7.06	<0.0001
	Survey2	-0.28	0.17	-1.69	0.09
Number of cells	Intercept	-2.24	0.23	-9.77	<0.0001
	Nests	0.09	0.02	4.57	<0.0001
Number of cells	Intercept	-1.52	0.09	-16.4	<0.0001
	Nests	1.32	0.27	4.97	<0.0001
	Nests2	-0.39	0.26	-1.51	0.13
